# Denervation Dynamics After Intramuscular BNT Injection in Patients With Focal Spasticity Monitored by MRI and Dynamometry–a Blinded Randomized Controlled Pilot Study

**DOI:** 10.3389/fneur.2021.719030

**Published:** 2021-11-19

**Authors:** Stefan Macher, Ewald Unger, Martin Zalaudek, Michael Weber, Gottfried Kranz, Georg Kranz, Gregor Kasprian, Thomas Sycha

**Affiliations:** ^1^Department of Neurology, Medical University of Vienna, Vienna, Austria; ^2^Center for Medical Physics and Biomedical Engineering, Medical University of Vienna, Vienna, Austria; ^3^Department of Biomedical Imaging and Image-Guided Therapy, Medical University of Vienna, Vienna, Austria; ^4^Neurologisches Rehabilitationszentrum Rosenhügel, Neurological Rehabilitation Center, Vienna, Austria; ^5^Department of Psychiatry, Medical University of Vienna, Vienna, Austria; ^6^Department of Rehabilitation Sciences, The Hong Kong Polytechnic University, Hong Kong, Hong Kong SAR, China; ^7^The State Key Laboratory of Brain and Cognitive Sciences, The University of Hong Kong, Hong Kong, Hong Kong SAR, China

**Keywords:** botulinumtoxin, denervation signs, magnetic resonance imaging (MRI), dynamometry, muscle denervation

## Abstract

**Introduction:** Botulinumtoxin associated muscle denervation (BNTMD) can be detected by magnet resonance imaging (MRI), MRI may provide further insights into the exact timeline of BNTMD and the potential impact and timing of physical exercise. We aimed to assess the time interval until detection of BNTMD by MRI and whether immediate physical exercise after intramuscular BNT injection has a measurable effect on clinical parameters and the intramuscular denervation dynamics illustrated by MRI.

**Materials and Methods:** Eleven age-matched patients were randomized to an “exercise” or “no-exercise” group. Eighty mouse-units of incobotulinumtoxin were injected into the spastic biceps muscle. MRI of the injected region, hand-held dynamometry of elbow flexor strength and clinical rating scales (mAS, CGI-I) were conducted in predefined intervals.

**Results:** We could not detect BNTMD within 24 h but 7 days after injection independent of group allocation (exercise *n* = 6, no-exercise *n* = 5). Denervation signs were more diffuse and spread into adjacent muscles in patients having received exercise. We could not detect differences concerning clinical measures between the two groups.

**Conclusions:** Physical exercise might influence BNTMD dynamics and promote propagation of T2-MR muscle denervation signs from the injected site into adjacent muscles.

**Trial registration:** clinicaltrialsregister.eu, Identifier 2017-003117-25.

## Introduction

After intramuscular injection botulinumtoxin (BNT) is internalized into nerve terminals at the motor endplate region, inhibits acetylcholine release and leads to a reduction in muscle tone. Magnetic resonance imaging (MRI) can visualize the local distribution of the injected BNT solution immediately after injection. Further, MRI can be used to depict BNT associated muscle denervation (BNTMD) by visualizing denervation associated muscle signal increase best detectable on fat saturated T2 weighted sequences (STIR). The intramuscular distribution of the toxin is dependent on the prevalent muscle architecture depicting different distribution patterns in spastic and healthy muscles when monitored by MRI (1). Previous results with heterogeneous observation periods indicated neurogenic denervation to occur earliest 3 months after BNT injection and persist until 12 months after baseline MRI (2, 3). BNTMD was not detectable by MRI 2 and 6 months after injection in a study that focused on muscle atrophy (4).

Muscle stimulation in context with intramuscular botulinumtoxin injection was shown to have a measurable clinical effect (5–7). BNT rapidly binds to SV2 receptors, is internalized by endocytosis and taken up continuously from the extracellular space in a step called diffusion (8). Experimentally it has been shown that in absence of nerve stimulation the translocation of the toxin may take <5 min and the lytic step (transmission of blockade) about 55 min (9). It has been shown in patients suffering from dystonia that repetitive stimulation initiated immediately after BNT injection led to significant reduction of compound muscle action potential (CAMP) measured by electromyogram (EMG) (6). We hypothesized that conducting physical exercise is rationale if done immediately before and after application probably leading to a measurable clinical effect in terms of reduction of muscle tone and strength level assessed by a hand-held dynamometer.

Within this pilot study we aimed to use MRI and dynamometry to examine the denervation dynamics after intramuscular BNT injection and the effect of exercise on the MR-detected denervation signals.

The primary objective of this study was to detect the time course of the MR-detectable intramuscular denervation signs after BNT injection. Additionally, we tried to figure out whether muscle exercise after BNT injection has measurable clinical effects and is reflected by different intramuscular denervation patterns in MRI studies.

## Materials and Methods

### Study Design

This was a prospective, randomized, controlled, and parallel-group pilot study. The protocol for this study was approved by the local ethical committee (EudraCT Nr.: 2017-003117-25).

### Inclusion Criteria

- 18 years of age
- suffering from upper limb spasticity with involvement of the biceps muscle
- a modified Ashworth Scale score of ≤ 2

### Exclusion Criteria

- existing myasthenic spectrum disorders, myotonia, myositis
- patients under anticoagulation
- pregnancy, breast-feeding
- presence of relevant metal parts or cardiac pacemakers

### Research Protocol

Twenty-two patients of 304 patients registered for botulinum toxin treatment in the database of the outpatient ward, Department of Neurology, Medical University of Vienna, matched the inclusion criteria according to existing medical records. Out of this sample 4 patients were recruited for randomization. Another 7 patients from the inpatient and outpatient ward of the department of neurology were recruited between 2017 and 2018. Overall, 51 patients were assessed for eligibility, 39 patients did not match the inclusion criteria, 1 patient declined to participate. Patients were randomized to the exercise-group (BNT injection+ elbow flexion) or non-exercise-group (BNT injection) by the principal investigator using the online tool “research randomizer.” Denervation edema illustrated by MRI, muscle strength measured by dynamometry and clinical scales were documented at predefined intervals (baseline, +1 day, + 7 days, + 4 weeks, + 8 weeks, + 3–7 months). Deviation from those intervals was tolerated if not > +/- 7 days. The unblinded principal investigator conducted the BNT injections and clinical measures (mAS, CGI-I, dynamometry). Radiological assessment was conducted by two blinded radiologists.

### Injection Protocol

Incobotulinumtoxin, delivered in a solid, pulverized state was diluted with 2 milliliters of saline solution and injected by a syringe holding a volume of 1 milliliter. For every patient a new box with an uncapped vial of incobotulinumtoxin was used. Patients received a standard dose of 80 MU BNT in the MBB within 4 h before baseline MRI. Injection was performed on two sites located +/- 2 cm from each other in the lower third of the MBB. During study participation no other upper arm muscle was treated. Additional muscles were injected according to clinical assessment.

### Exercise

Depending on the patient's individual ability to clench one's fingers or perform flexion in the elbow joint exercise contained either passive movement, active movement without additional weight or repetitive lifting of a 0.5L plastic water bottle. Elbow flexion was conducted 1 min before and 1 min after injection.

### Instruments

#### MRI

All MR examinations were performed on a 3T whole-body scanner (Achieva 3T, Philips Medical Systems, Best, The Netherlands). After tagging the injection site on the distal third of the upper arm with a MRI marker, study participants were positioned in the scanner in supine position with a flex surface medium coil positioned to the slightly adducted and inward rotated forearm, according to the tagged region of injection. Coronal STIR, axial T2 TSE and axial T1 DIXON TSE images were acquired for morphological and SI ratio evaluation (see Supplementary Table 1). Median values were calculated for measured Si-values. The T2 weighted signal intensity (Si) measured in the area of denervation was set in relation to normal muscle tissue of biceps muscle, triceps muscle and bone marrow. Out of these values three different quotients at each date of investigation were calculated: biceps.edema/biceps, biceps.edema/triceps, biceps.edema/bonemarrow. MRI data were crosschecked by two blinded radiologists with experience in neuromuscular imaging.

#### Dynamometry

Testing was conducted by use of a hand-held dynamometer (Sauter FK 500). Patients were sitting in the upright position with the shoulder in the neutral position and the upper arm in a slightly abducted position. The elbow was in positioned in a 90° angle, the wrist in supine position. The non-used arm was in a relaxed position, the legs were positioned parallel with floor contact. The wrist was positioned in a foam-lined clamp. The distal edge of the clamp was positioned over the cutaneous fold closest to the metacarpal bones. Distance from the cubital fossa to the middle of the clamp was measured and noted for each measurement. At first try patients were instructed to perform a single, low force level voluntary contraction to get familiar with the device. Each follow up testing was done without rehearsal. On examination patients were asked to increase elbow flexor force to the maximum level. The dynamometer was held with two hands by the investigator in a 90° angle to the patients arm with minimal deviation of +/-5° and counter pressure was exerted from above. Testing was stopped when resistance of patient's counterforce dropped. This exercise was repeated twice and the peak value of the patient's maximum force level was noted.

#### Clinical Scales

Modified Ashworth Scale Score (mAS) was assessed at baseline and at every follow up visit. Clinical Global Impression Scale (CGI-I) was assessed for each patient 4–8 weeks after injection and at final follow up visit. Both rating scales were administered by the same clinician over the whole study period. For CGI-I the patient's subjective assessment and clinicians rating were taken into account. If one was assessed twice within the observation period, the more favorable result was noted.

#### Statistics

Data analysis was performed using Microsoft Excel, version 16.34 and IBM SPSS Version 26. Descriptive statistics were calculated for all variables over four time points T0 = baseline, T1 = 7 days, T2 = 4–8 weeks, T4 = 3–7 months. Values were reported as median and interquartile range.

## Results

### Patients

**Figure 1:**
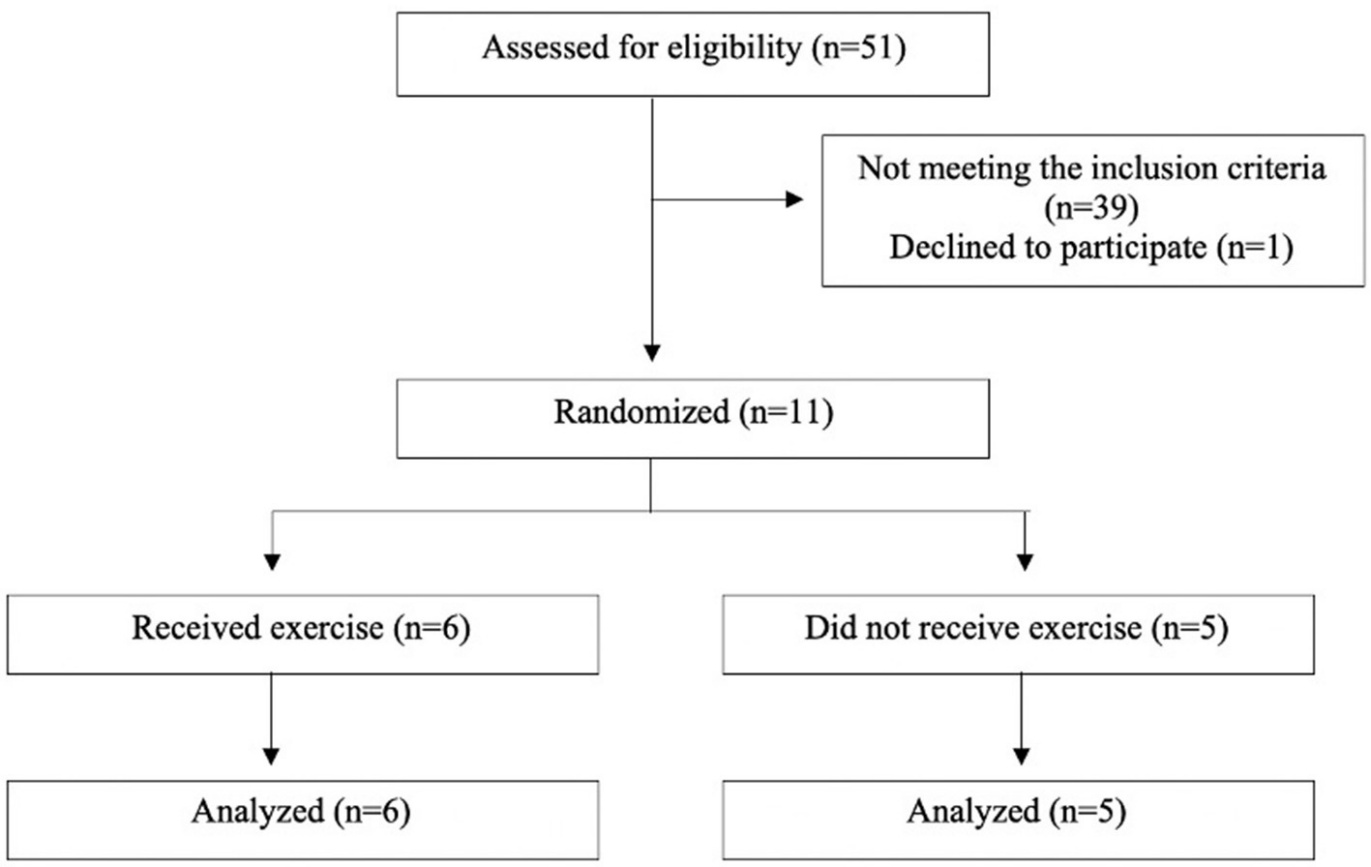
Flow chart of the study progress.

Details of the number of patients who were randomly assigned to the groups and completed the study are presented in Figure 1. For patient's individual details please refer to Table 1 and Supplementary Table 2.

**Table 1:** Summary of patient's individual data.

**ID**	**1**	**2**	**3**	**4**	**5**	**6**	**7**	**8**	**9**	**10**	**11**
**Sex**	**F**	**M**	**F**	**M**	**M**	**M**	**M**	**F**	**M**	**F**	**M**
**Age**	**71 y**	**70 y**	**48 y**	**74 y**	**72 y**	**68 y**	**62 y**	**52 y**	**51 y**	**58 y**	**73 y**
Etiology	Sub-arachnoidal hemorrhage	Ischaemic stroke	Spinal disc herniation	Intracranial hemorrhage	Ischaemic stroke	Ischaemic stroke	Ischaemic stroke	Ischaemic stroke	Ischaemic stroke	Ischaemic stroke	Ischaemic stroke
Clinical manifestation	Spastic mono-paresis	Spastic hemiparesis	Spastic paraparesis and monoparesis	Spastic tetraparesis	Spastic hemiparesis	Spastic tetraparesis	Spastic hemiparesis	Spastic hemiparesis	Spastic hemiparesis	Spastic hemiparesis	Spastic hemiparesis
Time since index event (months)	108 months	84 months	48 months	312 months	8 weeks	24 months	4 weeks	10 months	7 weeks	8 weeks	120 months
Time since last BNT injection	16 months	4 months	Naive	7 months	Naive	Naive	Naive	8 months	Naive	Naive	Naive
Injected muscles	MBB, MPT, FDS, FDP, FPB, MAP, FPL	MBB, MBR, MPT, FDP, FDS	MBB, FDP, FDS, MRF	MAB, MAL, MP, MVL, MVM, MBB, MBR, MS	MBB, MBR, FDS	MBB, MBR, FDS, FDP, FPL	MBB, MBR, FDS	MBB, FDS, FDP, MAP, MOP	MBB, MBR	MBB, MBR, FCR, FCU, FDS	MBB, MBR, FCR, FCU, FDS, FDP
BNT dose	275 MU	350 MU	230 MU	900 MU	140 MU	160 MU	130 MU	160 MU	120 MU	240 MU	300 MU
mAS baseline	2	2	1	2	1	1	1	1+	1	1+	2
Torque (Nm) baseline	4.86	13.13	7.99	9.12	11.73	9.73	8.42	9.92	15.6	7.04	12.68
Concomitant diseases	aHTN, HLP, depression	CAD, AF, CKD, DMTII	n.a.	Epilepsy, SSS	aHTN, HLP, epilepsy	Depression, carotid stenosis	DMTII, carotid stenosis	SVT, HLP	aHTN, HLP, depression	aHTN	aHTN, HLP, DMTII, Depression
Concomittant antispastic drugs	Baclofen	No	No	Tizanidine, Pregabaline, baclofen	No	No	No	No	No	No	No
Exercise	Yes	No	Yes	Yes	No	Yes	No	No	Yes	Yes	No
MRI follow up	8 days	3 months	7 months	1 month	7 days	1 month	5 months	3 months	4 months	5 months	5 months
PT during MRI follow up	No	Yes	No	Yes	Yes	Yes	YES	No	Yes	Yes	No
Adverse events	No	No	No	No	No	No	No	No	No	No	No

*CAD, coronary artery disease; aHTN, arterial hypertension; HLP, hyperlipidaemia; AF, atrial fibrillation; CKD, chronic kidney disease; DMTII, diabetes mellitus type II; SSS, Sick sinus syndrome; SVT, supraventricular tachycardia; PT, physiotherapy; MBB, M. biceps brachii; MPT, M. pronator teres; FDS, M. flexor digitorum superficialis; FDP, M. flexor digitorum profundus; FPB, M. flexor pollicis brevis; MAP, M. adductor pollicis; FPL, M. flexor pollicis longus; MBR, M. brachioradialis; MRF, M. rectus femoris; MAB, M. adductor brevis; MAL, M. adductor longus; MP, M. pectineus; MVL, M. vastus lateralis; MVM, M. vastus medialis; MS, M. supinator; MOP, M. opponens pollicis; FCR, M. flexor carpi radialis; FCU, M. flexor carpi ulnaris. Patients having received exercise are labeled gray*.

Across the groups 36% of patients were female and median age was 68 years. Age distribution between both groups was balanced (70 y no exercise vs. 63 y exercise) but the proportion of female patients was higher in the exercise-group (50 vs. 20%). The median time interval from index event to randomization was larger in the exercise-group (median [IQR]; 36 [7.5–93] vs. 10 [2–84]). Patients baseline torque level in the no-exercise-group was higher (median [IQR], 11.73 [9.92–12.86] vs. 8.56 [7.28–9.58]) and the median follow-up period was longer in the no-exercise group (median [IQR]; 90 [90–150] vs. 75 [30–142.5]). Participants in both groups were mainly BNT naive (4/6 exercise group vs. 3/5 no-exercise group). A third of patients in the exercise-group vs. no patient in the no-exercise-group used antispastic drugs. Patients received a median cumulative dose of 230 MU incobotulinumtoxin (min.-max.: 120 MU - 900 MU). Seven patients presented with a mAS Score of ≤ 1+ and four patients had a mAS-Score of 2 points. Seven patients (3 pat. in the exercise group) received physiotherapy at varying intervals and extent during the observation period. Concomitant antispastic drugs regimen did not change over the observation period. Patient 1 discontinued further study participation after 7 days due to personal reasons. We were not able to detect T2 denervation signals in this patient still we used the patient's dynamometry measures for analysis. Over the whole study period we did not observe any adverse events.

### Magnetic Resonance Imaging Intervals

MRI intervals were adapted according to imaging findings after two index patients (pat. 1 and 2) passed a follow-up period of 4 weeks. In both patients we could not detect T2 weighted muscle signal increase within 24 h after intramuscular BNT injection. Follow-up intervals were adapted according to these initial findings and set to baseline, +7 days, + 1 months, + 2 months, + 3 months, and + 4 months. Final MR-imaging > 4 months after injection was accepted if the patient did not receive repeated BNT.

With exception of one patient all 11 patients had at least 3 MRI follow ups. The shortest MRI follow up periods were 7 days in 3 patients, of those, patient 1 was excluded from MRI data analysis. Ninety-one percent of patients had at least 3, 36% had at least 4, 18% had at least 5 and 9% had 6 MRI follow ups (see Table 2 and Supplementary Figure 1).

**Table 2:** Quantitative data of MRI and dynamometry results in predefined follow up intervals.

	**Baseline/T0**				**7d/T1**				**4–8w/T2**				**3–7 mo/T4**			
**ID**	**Nm**	**Nm%**	**Si**	**Si%**	**Nm**	**Nm%**	**Si**	**Si%**	**Nm**	**Nm%**	**Si**	**Si%**	**Nm**	**Nm%**	**Si**	**Si%**
Pat.1^*^#	4.86	100			5.72	118										
Pat.2#	13.13	100	1.23	100	17.75	135	1.19	97	16.27	124	1.99	162	18.94	144	1.93	157
Pat.3^*^	7.99	100	1.14	100	8.55	107	1.76	107	9.50	119			9.21	115	1.64	144
Pat.4^*^#	9.12	100	1.63	100	7.64	84	1.47	90			1.87	115				
Pat.5	11.73	100	1.31	100	11.44	98	1.64	125	13.60	116						
Pat.6^*^	9.73	100	0.98	100	10.53	108	1.33	136	10.61	109						
Pat.7	8.42	100	1.01	100			0.96	95			1.24	123	18.88	224	1.34	133
Pat.8#	9.92	100	1.38	100			1.46	106	11.40	115	1.59	115			1.57	114
Pat.9^*^	15.60	100	1.27	100					12.54	80	1.56	115	16.50	106	2.36	186
Pat.10^*^	7.04	100	1.42	100					10.06	128	1.58	111			2.47	174
Pat.11	12.68	100	1.20	100					12.99	116	2.21	184			2.52	210
	2 MRI				3 MRI			4 MRI				5 MRI			6 MRI	

*colors indicate different numbers of follow-up MRI examinations; patients marked with*

* * were randomized to the exercise group; patients marked with # were pretreated with BNT. Si values refer to the quotient of the median value (Si) in the particular biceps edema zone divided by the median value (Si) in the corresponding area without edema in the biceps muscle. Nanometer (Nm)*.

### Timeline of BNTMD Assessed by MRI

We could not detect BNTMD within 24 h but 7 days after intramuscular BNT injection (see Supplementary Figure 2). The MRI dynamics detected 7 days after baseline were accompanied by patient reports on emerging clinical BNT effect recognized as increasing range of motion at elbow extension or reduced “catch” after repetitive use of the arm.

### The Effect of Exercise on MRI Parameters

A greater median maximum increase in intramuscular T2 Si was observed in the exercise group (54%, min.-max. 15–86%) compared to the no-exercise group (33%, min.-max. 15%-110%)-The median time to maximum Si alteration of the denervation zone was shorter in the exercise group (exercise 4 weeks, min.-max. 7 days−8 weeks vs. no exercise 8 weeks, min.-max. 7 days−5 months).

### The Effect of Exercise on Dynamometry

Over the whole study population torque levels increased in 3/11 patients, remained stable (+/- 20%) in 6/11 patients and decreased in 2/11 patients compared to baseline. No difference in change of torque level was observed between the groups (exercise median 14.5%, min.max.−16–143% vs. no-exercise median 16%, min.-max. 15–124%).

### Late Effects Visualized by MRI

In all patients with MRI follow up intervals > 12 weeks, Si values at last follow-up were higher than the previous imaging speaking for ongoing denervation dynamics even after weeks. The most marked increase in T2 signal alteration was observed in patients 2, 3, 9, 10, and 11, three of those patients received exercise (see Supplementary Figure 2). Still the effect of BNT was not reflected by dynamometric measurement which resulted in stable or increasing torque levels in all patients besides one.

### Denervation Patterns

Independent of group affiliation all patients showed T2 weighted muscle signal increase after intramuscular BNT injection. Over the course of the study period three different denervation patterns were noticed: (I) Early focal, injection-site-specific denervation signal following a rostro-caudal gradient evolving to extensive T2 weighted muscles signal increase restricted to one muscle belly, e.g. in patient 3, a sinusoidal course of T2 Si values was observed due to focal denervation edema distribution distal along the caput breve of the MBB (see **Figure 3A**). (II) A widespread denervation signal restricted to one muscle belly continuously increasing in T2 signal intensity over the course without further propagation (e.g., pat. 8) or (III) with propagation into the adjacent muscle belly (e.g., pat. 9 or 10).

Similarly to patient 2, findings concerning the restriction of denervation signs to one muscle belly were noted for patients 7 and 8 after BNT injection in the caput breve and caput longum of the MBB, respectively. Patient 10 showed a pre-existing feathery-like denervation pattern in baseline MRI which is known to may occur after muscle strain injury (10). Patient 2 was pretreated with BNT 4 months ago showing denervation signs at baseline which is reflected by quantitative data. Of note we found denervation signals restricted to the muscle belly injected with BNT in all patients in the no-exercise group vs. only one patient in the exercise group. In patients 9 and 10, both received exercise, T2 weighted intramuscular denervation signs were more diffuse and seemingly tended to spread to the opposite head of the MBB (see **Figure 3A**). The study did not focus on describing muscle alterations in the MBR therefore the muscle was not imaged in it's entirety and information out it's illustration is limited. However, in only half of the eight patients who received intramuscular BoNT injection into the MBR we could detect denervation signs (see **Figure 3B**).

Overall patient's satisfactory was rated good with a median CGI-I score of 3 points (IQR 1 p.). Despite patient's favorable rating of BNT effect by CGI-I, median mAS Score difference to baseline was 0p. (IQR 0.5 p.).

## Discussion

Within this pilot study we aimed to illustrate the longitudinal course of intramuscular denervation signs by MRI after BNT injections into the MBB and tried to narrow down the time interval until detection of an intramuscular denervation zone (EudraCT Number: 2017-003117-25, title: Longitudinal chemodenervation effects of botulinum toxin A after intramuscular application and the impact of physical activity). Additionally, we investigated if immediate exercise after BNT injection influences BNTMD and clinical parameters. Patients were divided into two groups and received physical exercise or not.

With our study we obtained several results: (I) The intramuscular distribution of the toxin early in the course was specific at the site of injection and seemingly the spread of the denervation zone was rather more localized in patients having not received exercise compared to a more diffuse distribution pattern and spread to the adjacent muscle belly of the MBB in patients having received exercise (see **Figure 3A**). (II) We could not detect T2 weighted intramuscular denervation signals within 24 h after BNT injection but found mildly to markedly increased T2 Si values at the injection side in 6/7 patients 7 days after baseline MRI (median increase 36%, min.-max. 25–54%). (III) The observed denervation signs may have possibly occurred earlier in the course than 7 days after baseline MRI, still the appearance of the denervation zone correlated well with the patients reported clinical effect of BNT action. (IV) An unexpected finding of the study is that in the majority of patients, regardless of group affiliation, an increase in elbow-flexor strength, mostly accompanied by a rise in T2 signal intensity in the injected biceps muscle was observed.

**Figure 2:**
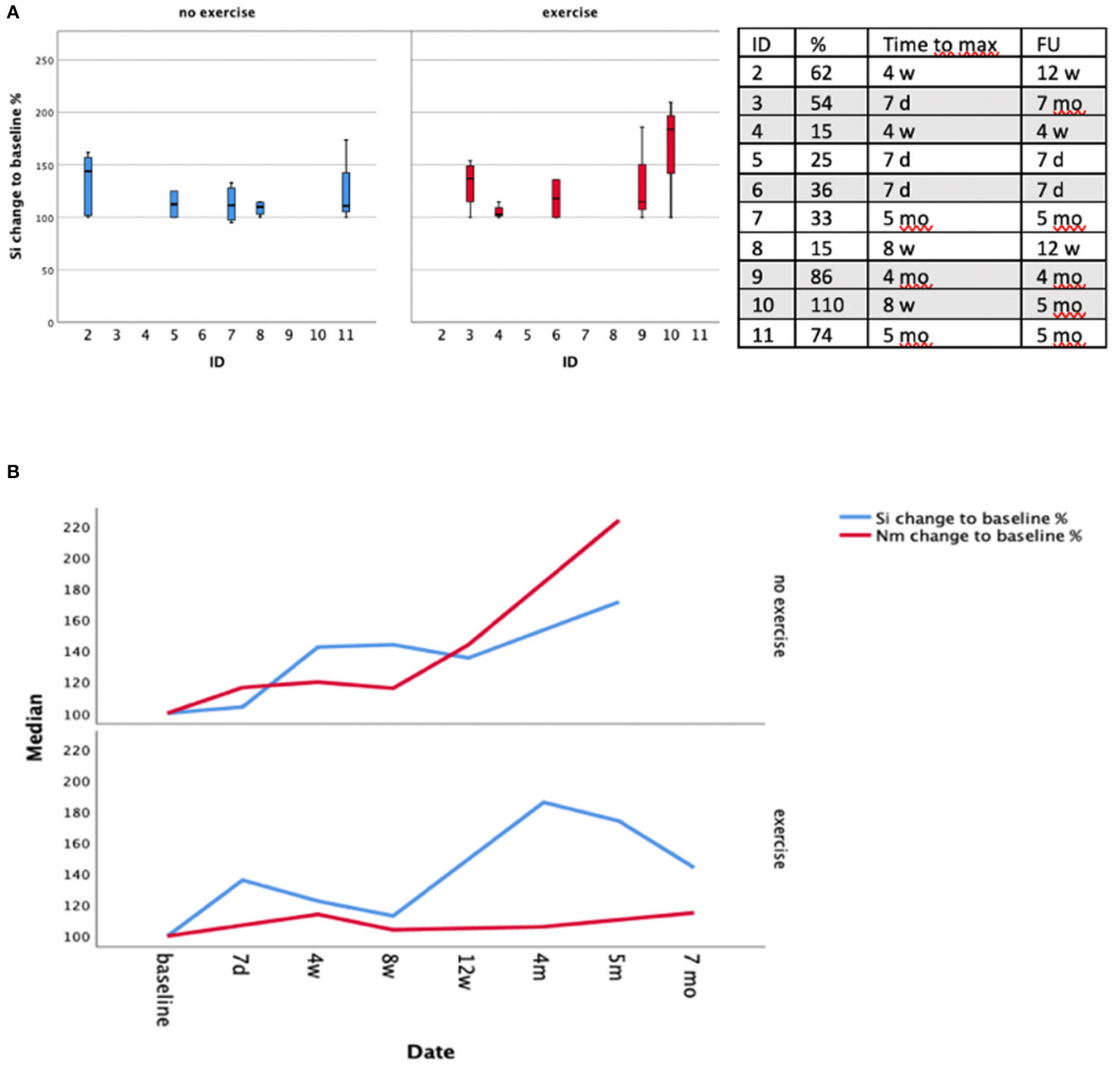
**(A)** Left: Longitudinal change in T2 signal intensity in the MBB with reference to baseline per patient. Right: Time interval until maximum T2 Si- value was reached per patient. Patients having received exercise are labeled gray **(B)** Median change in strength level (Nm, %) and T2 signal intensity (Si, %) compared to baseline levels across the groups (exercise vs. no exercise).

This study might have several relevant clinical implications to be considered in the rehabilitation process. First, our results indicate that exercise, if conducted immediately before and after intramuscular BNT injection might lead to a more widespread distribution pattern of the denervation zone into close-by muscles. In our small collective this was rather observed in patients having received exercise showing dispersion of the denervation signal into the adjacent muscle belly of the MBB opposite to the injection site. Still, in patients 4 and 6 of the exercise group we could not detect widespread denervation pattern. Of note MRI follow up period was only 28 days in these patients and we cannot exclude similar denervation patterns might have occurred later in the course. Overall, there was no detectable difference concerning strength levels in patients with extensive denervation signs and/or higher cumulative BNT doses (> 200 MU) compared to patients with local denervation signs and/or lower BNT doses (<200 MU). In the majority of our patients, regardless of group affiliation, stable or increasing elbow-flexor strength, mostly accompanied by a rise in T2 signal intensity in the injected biceps muscle was observed over the study period (see Figure 2 and Supplementary Figure 2). Assuming that exercise leads to extension of the denervation signal in adjacent muscles one would expect a stronger clinically measurable BNT-effect after physical exercise. If this is reflected by clinical scales and patient's subjective evaluation or if this might have dose-saving implications need to be proven by larger studies.

Unexpectedly we found loss of elbow flexor strength only in two patients (4 and 9). Observation period was rather short in patient 4, and patient 9 showed loss of strength 8 weeks after injection which was regained after BNT effect subsided. All other patients had more or less stable strength levels whereas three patients (2, 7, 10) showed increasing levels of at least ca. 1/3 compared to baseline measurement over the course of the observation period. Gain of strength after intramuscular BNT injection is usually admitted to loss of spasticity in the injected muscle and consecutively improved flexibility in the involved joint leading to reinforcement of antagonistic muscles. However, this effect does not apply to our collective. Our results are supported by findings from a previous study reporting increasing grip strength after BNT injection in ca. 2/3 of patients and increasing elbow flexion strength in ca. 40% of patients which was accompanied by a significant decrease in flexor EMG amplitude (11). The authors account the reduction of motor units as a possible economization factor on the one hand and facilitation of upper arm muscle function due to gain of strength in grip and elbow flexion for this effect on the other hand. Eventually there is an age-dependent effect of lower numbers and/or sizes of motor units associated with higher strength levels but if this leads to higher torque levels after intramuscular BNT injection is speculative (12). Yet the reduction in the number of motor units which is observed in aging people is not necessarily accompanied by a loss of strength (13).

Of note in some patients (5, 7, 9, 10) the time interval from index event to baseline examination was ≤ 8 weeks. A “natural course” of regaining strength after cerebral stroke in combination with physiotherapy might account for increasing strength levels in this group of patients. This is especially true for pat. 7 and 10 and patients generally suffered from mild to moderate spasticity (mAS Score <2) prior to BNT injection. Tone reduction and possibly better utilization of the extremity in activities of daily living—in terms of increased level of exercise—might also have contributed to a gain in strength levels (for patient's individual data please refer to Figure 2 and Supplementary Figure 2).

Few studies focused on intramuscular denervation dynamics after BNT injection. Van Campenhout et al. conducted MRI studies following intramuscular BNT injections into the psoas muscle of children with cerebral palsy. MRI was conducted 2 and 3 months after BNT injection (earliest 57 days) and muscle atrophy was found in all patients whereas a clear denervation zone was not detectable though the study was designed to focus on volumetric measures (4). Fanucci et al. demonstrated muscle atrophy accompanied by rising signal intensity values assessed by MRI earliest 3 months after intramuscular BNT injection into the piriformis muscle (3). In that respect Schroeder et al. confirmed muscle atrophy and increasing T2 signal intensity values earliest 3 months after injection of BNT into the gastrocnemius muscle. These studies do not provide information on MRI dynamics the first few days after intramuscular BNT injection, still they revealed that muscle atrophy is accompanied by rising T2 signal intensity values (14). In an experimental animal study Wessig et al. showed that MR-denervation signs were detectable as soon as 24 h and latest 48 h after denervation. Denervation signs in human musculature can be detected by MRI earliest 4 days after the onset of the denervation-triggering event whereas MR-atrophy occurs as early as several weeks after denervation (15). Our results indicate that the occurrence of an intramuscular denervation zone detectable by MRI may be expected between 24 h and 7 days after BNT injection. Incobotulinumtoxin is delivered in a solid, pulverized state and was diluted with 2 milliliters of saline solution prior to intramuscular injection. The BNT-saline solution can be detected by muscle-MRI immediately after injection. Within minutes distribution along the parallel muscle fiber architecture of the biceps muscle and absorption of the solution by muscle cell tissue was observed (1). We could not detect an intramuscular fluid layer nor signs of T2 weighted denervation by MRI 24 h after BNT injection. Hence, we conclude that intramuscular T2 Si increase detected 7 days after BNT injection were not mis-constructed as denervation signs as the injected solution would have probably been absorbed entirely at that time. Beyond that the increasing T2 weighted muscle signal increase over the time course in our patients is rather attributable to a denervation process than distribution of the BNT-saline solution.

**Figure 3:**
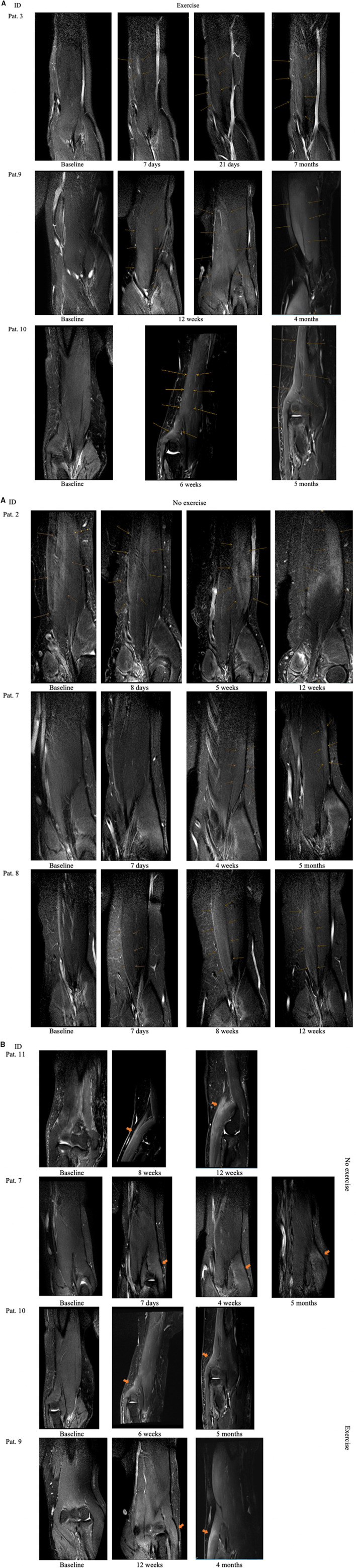
**(A)** Denervation sign after intramuscular BNT injection in the MBB of patients allocated to the exercise group and no-exercise group. Pat. 2 was pretreated with BNT. arrows indicate area of denervation. **(B)** Denervation signs in patients *M. brachioradialis*.

This study has some important limitations. A possible effect of the single-blinded study design on the obtained results cannot be excluded. Some patients showed a feathery-like denervation pattern at baseline examination independent of BNT pretreatment status. We found preexisting T2 signal alterations in 5/7 patients with chronic spasticity (defined as > 8 weeks from index event) which imposed more or less as a feathery-like pattern. Four of those patients were pretreated with BNT. A clear-cut distinction between preexisting denervation signs due to chronic spasticity and/or BNT treatment could not be drawn. Patient 2 suffered from chronic spasticity and received his last BNT injection in the MBB 4 months prior to baseline MRI which was probably targeted to the caput breve of the MBB (see Figure 3A). The preexisting denervation sign which is visible at baseline examination and 8 days after (see arrows Figure 3A) diminished markedly concerning T2 signal intensity and expansion over the course speaking for BNT-induced denervation pattern. Still morphological, supported by our quantitative data, this should not have influenced our findings significantly. We found focal T2 denervation signals in the MBR of patients but could not detect specific MRI patterns according to group affiliation. This may be due to the small size of the muscle. However, not all patients received BNT injections into the MBR and the study was not focused on imaging this muscle. Our study lacks a control group and we did not image the contralateral BNT untreated arm to support our findings. Concomitant antispastic drugs remained unchanged but some patients received unstandardized physiotherapy sessions during the study period which was balanced between the groups. A “natural course” in four patients who also received physiotherapy and were included soon after the index event might explain the gain of strength in at least two patients (7 and 10). The small sample size and the heterogeneous individual observation periods are a major limitation of this study and therefore significance of the obtained results is limited. Still this is a pilot study without existing data on the expected effect size and the inter-individual variability. Further adherence to predefined follow up dates, making an appointment and alignment with available MRI slots turned out to be challenging. We used a hand-held dynamometer for measurement of isometric muscle strength and did only note the maximum elbow flexor strength level at each follow up interval. The use of an electro-goniometer simultaneously measuring the range and strength of movement in a longitudinal design would have delivered more detailed results. Nevertheless, the use of a hand held device showed good reliability and validity before (16).

We conclude that MR-associated denervation signs tend to appear between 24 h and 7 days after intramuscular BNT injection. Physical exercise conducted immediately before and after intramuscular BNT injection might influence denervation dynamics as illustrated in the MBB by MRI in our study. Whether immediate exercise before and/or after BNT injection influences convection or diffusion of the toxin is a matter of debate. Spreading of denervation signals into adjacent muscles could lead to a greater clinical effect in terms of loss (strength) or gain (spasticity) of function whereby dose-saving effects might become relevant. On the other hand, abandonment of exercise could be important if e.g., only the caput longum of the MBB shall be injected in order to prevent dislocation of the shoulder. Our findings cannot be applied one to one to clinical routine but need to be verified by blinded, larger sample-size trials with an additional placebo group.

## Data Availability Statement

The raw data supporting the conclusions of this article will be made available by the authors, without undue reservation.

## Ethics Statement

The studies involving human participants were reviewed and approved by Ethics Committee of the Medical University of Vienna. The patients/participants provided their written informed consent to participate in this study.

## Author Contributions

SM had full access to all of the data in the study and takes responsibility for the integrity of the data and the accuracy of the data analysis. SM and TS: concept and design and drafting of the manuscript. SM and MW: statistical analysis. All authors: acquisition, analysis, interpretation of data, and critical revision of the manuscript for important intellectual content.

## Conflict of Interest

SM has participated in meetings sponsored by, received speaker honoraria or travel funding from Merz. GK has participated in meetings sponsored by, received speaker honoraria or travel funding from Merz, Ipsen, and Allergan. TS has participated in meetings sponsored by, received speaker honoraria or travel funding from Merz, Ipsen, and Allergan. The remaining authors declare that the research was conducted in the absence of any commercial or financial relationships that could be construed as a potential conflict of interest.

## Publisher's Note

All claims expressed in this article are solely those of the authors and do not necessarily represent those of their affiliated organizations, or those of the publisher, the editors and the reviewers. Any product that may be evaluated in this article, or claim that may be made by its manufacturer, is not guaranteed or endorsed by the publisher.
