# Supplementary material for: Denervation Dynamics After Intramuscular BNT Injection in Patients With Focal Spasticity Monitored by MRI and Dynamometry–a Blinded Randomized Controlled Pilot Study
Source: Front Neurol. 2021 Nov 19;12:719030. doi: 10.3389/fneur.2021.719030 (PMC8640502; doi:10.3389/fneur.2021.719030)
Supplement: Supplementary file 1 [file Table_1.docx]

|  | **STIR cor** |  | **2D T2 ax** |  | **T1 DIXON ax** |  |  |
| --- | --- | --- | --- | --- | --- | --- | --- |
|  | **IR** |  | **TSE** |  | **TSE** |  |  |
| TE [ms] | 55 |  | 100 |  | 7 |  |  |
| TR [ms] | 5565 |  | 4785 |  | 621 |  |  |
| TI [ms] | 200 |  | - |  | - |  |  |
| Matrix | 364 x 271 |  | 259 x 247 |  | 131 x 127 |  |  |
| FOV read [mm] | 200 |  | 130 |  | 131 |  |  |
| FOV phase [mm] | 154 |  | 108 |  | 109 |  |  |
| No. slices | 25 |  | 40 |  | 46 |  |  |
| Voxel size | 0.55 x 0.74 x 2.5 |  | 0.5 x 0.53 x 3.5 |  | 1 x 1.03 x 2.0 |  |  |
| FA [°] | 90 |  | 90 |  | 90 |  |  |
| Fat saturation | - |  | no |  | - |  |  |
| Bandwidth (Hz/px) | 187 |  | 218 |  | 810 |  |  |
| TA [min:s] | 2:16 |  | 2:15 |  | 2:15 |  |  |
| STIR, Short Tau Inversion Recovery; 2D T2 TSE, 2-dimensional T2-weighted turbo spin echo; T1 DIXON TSE, T1-weighted DIXON with turbo spin echo; TE, echo time; TR, repetition time; FOV, field of view; FA, flip angle; TA, acquisition time | | | | | | | |

Supplementary Table 1 MRI Sequence parameters
