# Supplementary material for: Denervation Dynamics After Intramuscular BNT Injection in Patients With Focal Spasticity Monitored by MRI and Dynamometry–a Blinded Randomized Controlled Pilot Study
Source: Front Neurol. 2021 Nov 19;12:719030. doi: 10.3389/fneur.2021.719030 (PMC8640502; doi:10.3389/fneur.2021.719030)
Supplement: Supplementary file 2 [file Table_2.docx]

Supplementary Table 2 Demographic details

|  | Exercise | No exercise |
| --- | --- | --- |
| Sex (female; %) | 50 | 20 |
| Age (y), median (IQR) | 63 (17.5) | 70 (10) |
| Time since index event (months), median (IQR) | 36 (85.5) | 10 (82) |
| Dynamometry baseline (Nm), median (IQR) | 8.56 (2.3) | 11.19 (2.94) |
| BNT naive (%) | 66,6 | 60 |
| Antispastic drugs (%) | 33,3 | 0 |
| Etiology (ischemic; %) | 50 | 100 |
| MRI follow up (days), median (IQR) | 75 (112.5) | 90 (60) |
| Number of MRI during follow up | 3,7 | 3,4 |
| Physiotherapy during follow up | 66,6 | 60 |
