# Supplementary figures and images for: Denervation Dynamics After Intramuscular BNT Injection in Patients With Focal Spasticity Monitored by MRI and Dynamometry–a Blinded Randomized Controlled Pilot Study
Source: Front Neurol. 2021 Nov 19;12:719030. doi: 10.3389/fneur.2021.719030 (PMC8640502; doi:10.3389/fneur.2021.719030)

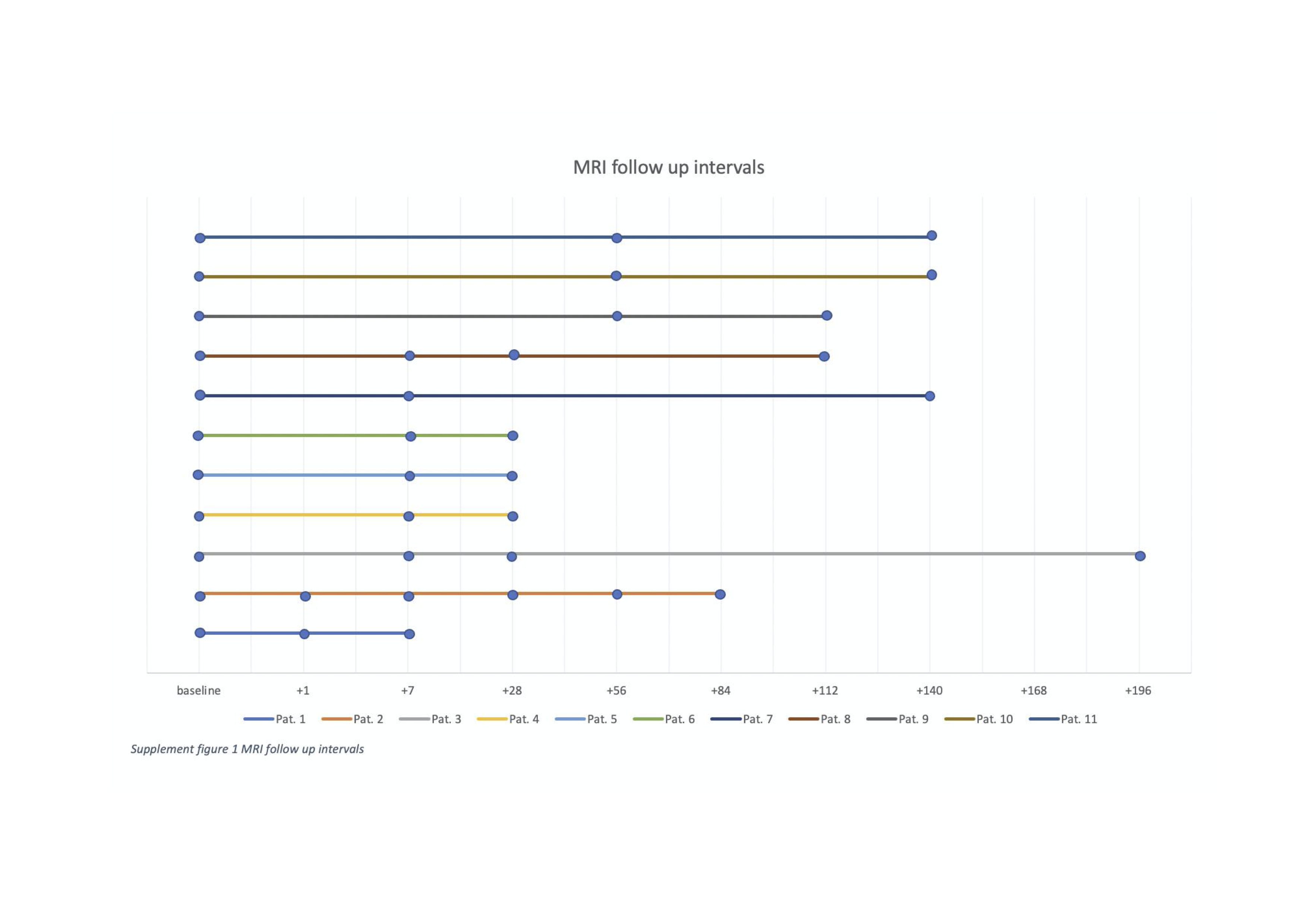

Supplement: Supplementary file 3 [file Image_1.tiff]

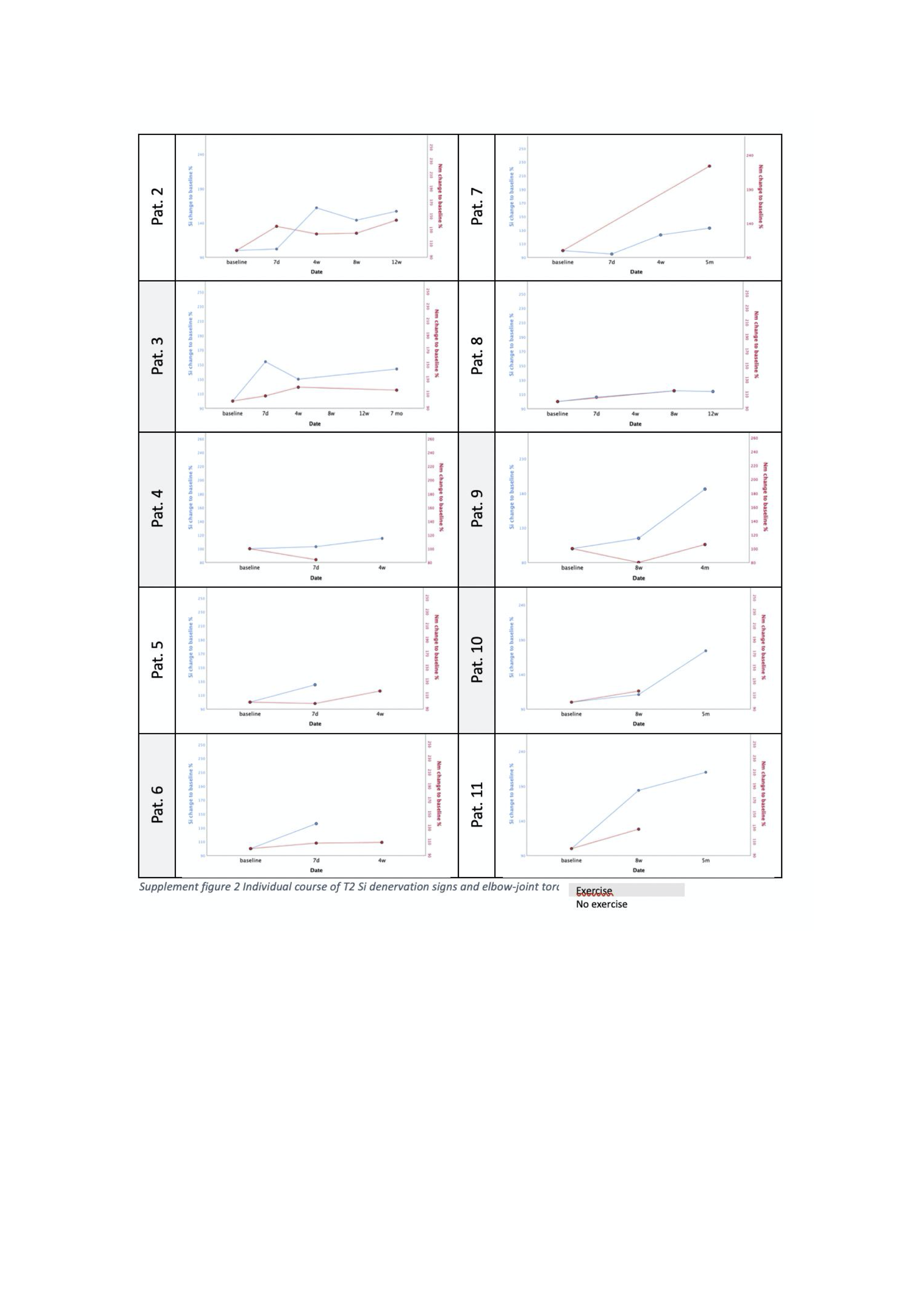

Supplement: Supplementary file 4 [file Image_2.tiff]
